# Development and application of a qualitative rapid analysis framework in a hybrid trial within primary care

**DOI:** 10.1136/bmjopen-2023-076792

**Published:** 2024-07-24

**Authors:** Amy Mathieson, Rebecca Elvey, Paul Wilson

**Affiliations:** 1 Centre for Primary Care and Health Services Research, Division of Population Health, The University of Manchester, Manchester, UK

**Keywords:** Primary Care, QUALITATIVE RESEARCH, Patient Participation, Implementation Science, Clinical Trial, STATISTICS & RESEARCH METHODS

## Abstract

**Context:**

In the context of iterative feedback loops to support real-time policy decision making, and an emphasis on speeding up adoption of evidence-based interventions, qualitative healthcare researchers are increasingly expected to produce rapid results and products. Traditional qualitative methods have been adapted for this purpose.

**Objective:**

To develop and apply a rapid analysis framework in a process evaluation for the VICTORION-Spirit study; a ground-breaking hybrid trial examining real-world delivery of inclisiran—a cholesterol-lowering treatment—in primary care.

**Design:**

We developed a rapid analysis framework, using a summary template, to analyse data from semistructured telephone interviews.

**Setting:**

Primary care in Greater Manchester, UK.

**Participants:**

Patients who had received inclisiran as part of the VICTORION-Spirit trial (56), providers delivering inclisiran (28) and representatives from the Academic Health Science Network (8) participated in the original study.

**Results:**

The rapid analysis framework we developed and applied comprised six steps: (1) creating a summary template based on the five Consolidated Framework for Implementation Research domains; (2) test-driving, refining and finalising the summary template; (3) completing the template soon after each interview using field notes; (4) discussing analysis as a team; (5) transferring summaries to a matrix; and (6) using the summary matrix to inform presentations and interim reports for stakeholders. Our rapid analysis framework saved time and improved efficiency, as we were able to feedback barriers to stakeholders in real time via presentations.

**Conclusions:**

Rapid analysis in applied healthcare research can produce timely and trustworthy findings. Our rapid analysis framework would be useful within studies where there is a need to feedback to stakeholders and adjust implementation strategies accordingly in real time. Thus, supporting successful implementation efforts and accelerating adoption.

**Trial registration number:**

NCT04807400, 19/03/2021.

**Strengths and limitations of this study**

- Our study explored a new rapid analysis framework, using a summary template, in a hybrid trial to ensure the timely reporting of trustworthy findings to clinicians, trial staff and policy makers.
- The work describes the process of developing and applying our rapid analysis framework, and our experience of analysing and sharing findings on implementation challenges in real time, to help others plan future use.
- Due to time constraints, we did not compare our rapid analysis framework with a traditional qualitative analysis approach such as thematic analysis.
- Our study shows it is possible to conduct rapid analysis within the context of rigorous evaluation.

## Background

 Qualitative methods are used to help understand implementation and the effects of innovations within real-world conditions and populations. In a public health service context, which is often subject to budget cuts and policy changes, the usefulness of these research findings may depend on how quickly they are shared. Traditionally, qualitative research can require long periods of time for data collection and analysis. This can be particularly challenging where researchers need to provide real-time data to inform implementation processes or understand the factors that may help or hinder implementation of evidence-based interventions. Consequently, there is a need for rigorous rapid research designs.^1^

Over the last three decades, researchers have developed rapid research design approaches to speed up data collection, analysis and dissemination such as Rapid Assessment Process,^2^ Rapid Qualitative Inquiry^3^ and Rapid Ethnography.^4^ Several approaches to conducting rapid qualitative evaluations, inquiries or assessments have been outlined in the literature.^5^,^10^ While there is currently a lack of consensus on what is considered ‘rapid’, with suggestions ranging from a few days to 6 months,^11^ most rapid research approaches have these features in common: (a) the study is conducted in a short time frame, (b) the study is a team-based inquiry, (c) it uses multiple research methods and triangulation, and (d) data collection and analysis are iterative.^3 10^

Researchers have also developed techniques to save time, which can be incorporated into rapid qualitative research designs or be used in longitudinal research, including reducing data collection time; directly analysing audio recordings; summarising data instead of formal coding, using mind maps, notes or a ‘one page summary’; and using voice recognition software to avoid manual transcription. In their recent systematic review exploring the ways in which qualitative methods have been adapted to save time, Vindrola-Padros and Johnson^12^ found more than half of the 18 included studies described rapid techniques for data analysis. Most of these studies relied on verbatim transcripts,^13 14^ directly coded interview audio^15^,^18^ or used voice recognition software to avoid manual transcription.^19^,^21^

Several benefits to using rapid research techniques have been described in the literature.^12^ Most authors reported a reduction in time between data collection and analysis, which has been particularly useful in response to infectious pandemics,^22^ and when findings are needed to quickly adjust implementation strategies and update stakeholders. The time saved also enables more data collection, the inclusion of more research participants, and reduces costs. Rapid techniques may also reduce interpretative bias when creating an interview transcript, limit human error, provide greater insight into the data and better links to theory, and enhance transparency of the data analysis process.^12^ However, rapid techniques are not without limitations, and some authors have discussed challenges around achieving the same level of interpretation as traditional methods, the need to be selective and possibly ‘losing data’,^12^ the risk of researcher bias, and maintaining trustworthiness.^10^ Further, other literature has reported heavy workloads due to the short time frames,^23^ and the cost and additional time needed to master new technologies for example voice recognition software.^12^

Despite these limitations, rapid research techniques are increasingly being used in health services, and implementation, research. Hamilton developed a rapid qualitative analysis approach that summarises interview transcript data into a template, which is then condensed into a matrix.^24^ Gale *et al*^14^ developed this method, into one that was informed by theory, by incorporating the Consolidated Framework for Implementation Research (CFIR); a determinants framework, widely used in implementation research to guide the systematic assessment of barriers and enablers to adoption and spread of innovations.^25^ Gale et al’s approach involves summarising transcript data into a template and then mapping themes onto the CFIR domains. We conducted a CFIR-informed rapid analysis, based on Hamilton’s and Gale et al’s approaches. In this article, we describe the tool and technique we developed and applied in the process evaluation for the hybrid trial outlined below.

### Context and aim of the study

Recently, health policy in England has emphasised innovation, speeding up the adoption and uptake of new products, and closer working between the NHS and industry. In 2016, the Accelerated Access Review called for efforts to develop a more streamlined pathway to identify, develop, approve and adopt high-value innovations in the NHS. The Review recommended establishing an Accelerated Access Pathway for streamlining regulatory, reimbursement, evaluation and diffusion processes, to bring products to patients quicker.^26^ Recommendations included the development of the NHS Accelerated Access Collaborative (AAC), which aims to provide faster access to innovations in the NHS by addressing barriers to real-world adoption.^27^

Inclisiran—a new cholesterol-lowering treatment—is one of the first products fast-tracked by the NHS AAC to be deployed at scale and in tandem with a streamlined pathway for regulatory approval and technology appraisal by the National Institute for Health and Care Excellence.^28^ Whereas statins are taken as tablets daily, inclisiran is administered by subcutaneous injection, two times per year. The safety and efficacy of inclisiran were evaluated through the Phase III ORION clinical development programme, comprising three randomised control trials: MDCO-PCS-17–03 (ORION-9), MDCO-PCS-17–04 (ORION-10) and MDCO-PCS-17–08 (ORION-11).^28^ Inclisiran has been made available via the NHS Population Health Agreement between NHS England and NHS Improvement, the Academic Health Science Network (AHSN), and Novartis Pharmaceuticals UK Limited. The VICTORION-Spirit study was conducted to help understand how a new technology could be deployed and delivered into a system of care in a way that would treat patients at a population-health level; to accelerate patient access and improve patient outcomes.^29^

Greater Manchester was the setting for the VICTORION-Spirit study (Trial Registration: NCT04807400, 19/03/2021). The VICTORION-Spirit study is a ground breaking type 1 hybrid trial, which aimed to provide and assess evidence for the implementation of inclisiran within a primary care setting. In brief, VICTORION-Spirit is a phase IIIb, multicentre, open-label, randomised controlled trial, in which 900 patients on established cholesterol-lowering medication were randomised to one of three groups: (1) usual care (eg, statins or ezetimibe) plus behavioural telephone support, (2) usual care plus inclisiran injections, and (3) usual care plus inclisiran injections and behavioural telephone support.

As part of this study, our team conducted the VICTORION-Spirit process evaluation to understand what is needed to support the implementation of inclisiran in the real world. Drawing on the CFIR, we aimed to explore the feasibility and acceptability of inclisiran delivery in primary care to patients and professionals, and wider ‘transactability’—how to organise, deliver and maintain the innovation. The CFIR comprises five domains likely to influence the implementation of complex interventions: (i) intervention characteristics, (ii) outer setting, (iii) inner setting, (iv) characteristics of individuals, and (v) process. Each of the five domains is further divided into 39 constructs, which have been associated with effective implementation.^25^

To ensure relevance to the needs of the NHS, AAC and AHSN and to maximise impact, we sought to provide these key stakeholders with timely insights from the process evaluation as they emerged over the course of the study. To support this need to inform real time policy decision-making and national implementation, we adopted a qualitative approach using rapid analysis.

The objective of this article is to describe how we developed, applied and used a rapid analysis summary template. We will also reflect on our experience of analysing and sharing findings on implementation challenges in real time and discuss what this may mean for future qualitative health services research.

## Method

### Process evaluation design and data collection

**Figure 1:**
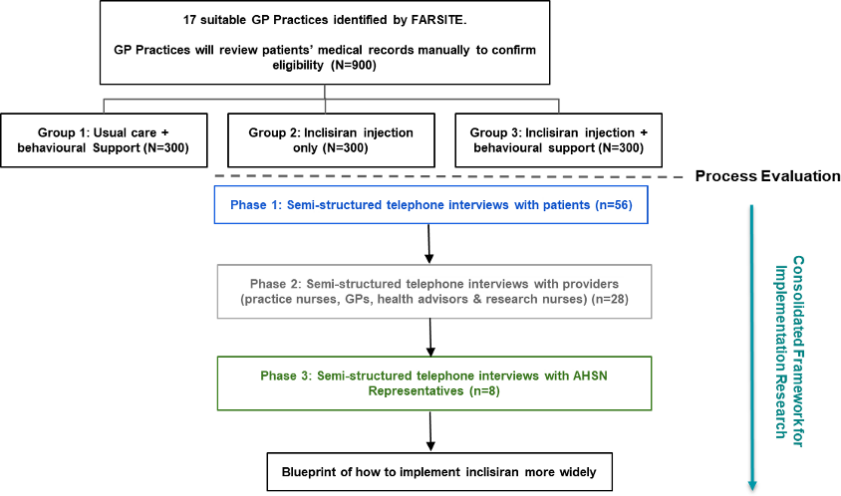
Process evaluation data collection flowchart.

The process evaluation had three phrases (figure 1), and aimed to:

1. Explore the views and experiences of those delivering, and receiving, inclisiran.
2. Identify barriers and enablers to integrating delivery models for inclisiran within primary care.
3. Identify the ‘core enabling ingredients’ to support the wider delivery of inclisiran within the NHS.

Our rapid analysis approach was developed for analysing the qualitative data from interviews with patients, providers and AHSN representatives. Interviews were semi-structured and conducted by two researchers (AM and RE). All authors are experienced researchers. AM and RE are trained to PhD level and have experience of the process of recruiting, conducting interviews and analysing data, gained in a variety of healthcare and community settings. Interview topic guides—one for each participant group—were designed to incorporate the five CFIR domains (ie, intervention characteristics, outer setting, inner setting, characteristics of individuals and process) (box 1). Data from the three participant groups were collected concurrently.

Box 1 **Topics covered in interviews**

**Interviews with patients explored their views and experiences of:**

- Managing high cholesterol.
- Taking part in the trial.
- Receiving the injection and attending the appointments, and where relevant, experience of the behavioural support programme.

**Provider interviews explored:**

- The structures, resources and processes required to embed inclisiran into routine general practice.
- Any challenges to implementation.
- Patients’ response to inclisiran.
- Their views on future provision.

**Interviews with AHSN representatives explored:**

- Their role in the national lipids management optimisation programme.
- Their understanding of the role of inclisiran within the lipid management pathway.
- How the AHSN engages and supports stakeholders with regard to inclisiran.
- Local drivers and barriers to the roll out of inclisiran.
- Their views on the sustainability of current delivery models.

Participants were purposively sampled. The inclusion criteria were people receiving the treatment, or involved with provision of the service as providers, facilitators or commissioners. Patients were recruited via telephone invite, and providers and commissioners were recruited via email. Interviews with patients were conducted via telephone. Provider participants were given the choice of interview mode (via video conferencing or telephone). Most opted for a telephone interview and two were interviewed via MS Teams. Commissioner interviews were conducted on MS Teams. Verbal consent was confirmed with all participants and audio-recorded separately before the interviews.

All interviews were audio-recorded, and later, transcribed for future analysis. The study was reviewed and approved by the Health Research Authority in England (IRAS: 289136) and South Central - Berkshire Research Ethics Committee (reference number: 29136).

### Patient and public involvement

Patients and/or the public were not involved in the design, conduct, reporting or dissemination plans of this research.

### Our approach to rapid analysis

After reviewing the literature, we developed a rapid analysis technique based on Gale et al’s adaption of Hamilton’s rapid qualitative analysis approach.^14^ The rapid analysis (RA) technique and tool we developed and applied is summarised in box 2 and comprised the following steps:

Box 2 **Summary of our rapid analysis framework**

Step 1: Creating a summary template

Step 2: Test-driving, refining and finalising the summary template

Step 3: Completing the template soon after each interview

Step 4: Discussing analysis as a research team

Step 5: Transferring summaries (step 3) to a matrix

Step 6: Using the summary matrix to share findings

### Step 1: creating a summary template

The first step of our RA approach involved developing a template, which the team would use to summarise each interview. To create the summary template, AM and RE reviewed the CFIR-informed interview guides and constructed a table in MS Word. The table had two columns. The first column specified CFIR domains and constructs (eg, ‘Intervention characteristics - advantageous compared with just taking statins’), interview questions (eg, ‘Context’ - how long has the patient had high cholesterol?), and barriers and facilitators. The second column was blank and used to summarise key data from the interviews that corresponded to the CFIR constructs. So as not to overly rely on CFIR, we also included space for other observations or unexpected findings to highlight issues that emerged inductively. Lastly, we included a ‘key quotations’ box, designed to capture illustrative verbatim or paraphrased quotes from the interviews. The overall aim in designing the template was to enable a summary of aspects of the implementation of inclisiran, including barriers and enablers. We drafted three templates, one for each participant group: patients, providers and commissioners.

### Step 2: test-driving, refining and finalising the summary template

**Figure 2:**
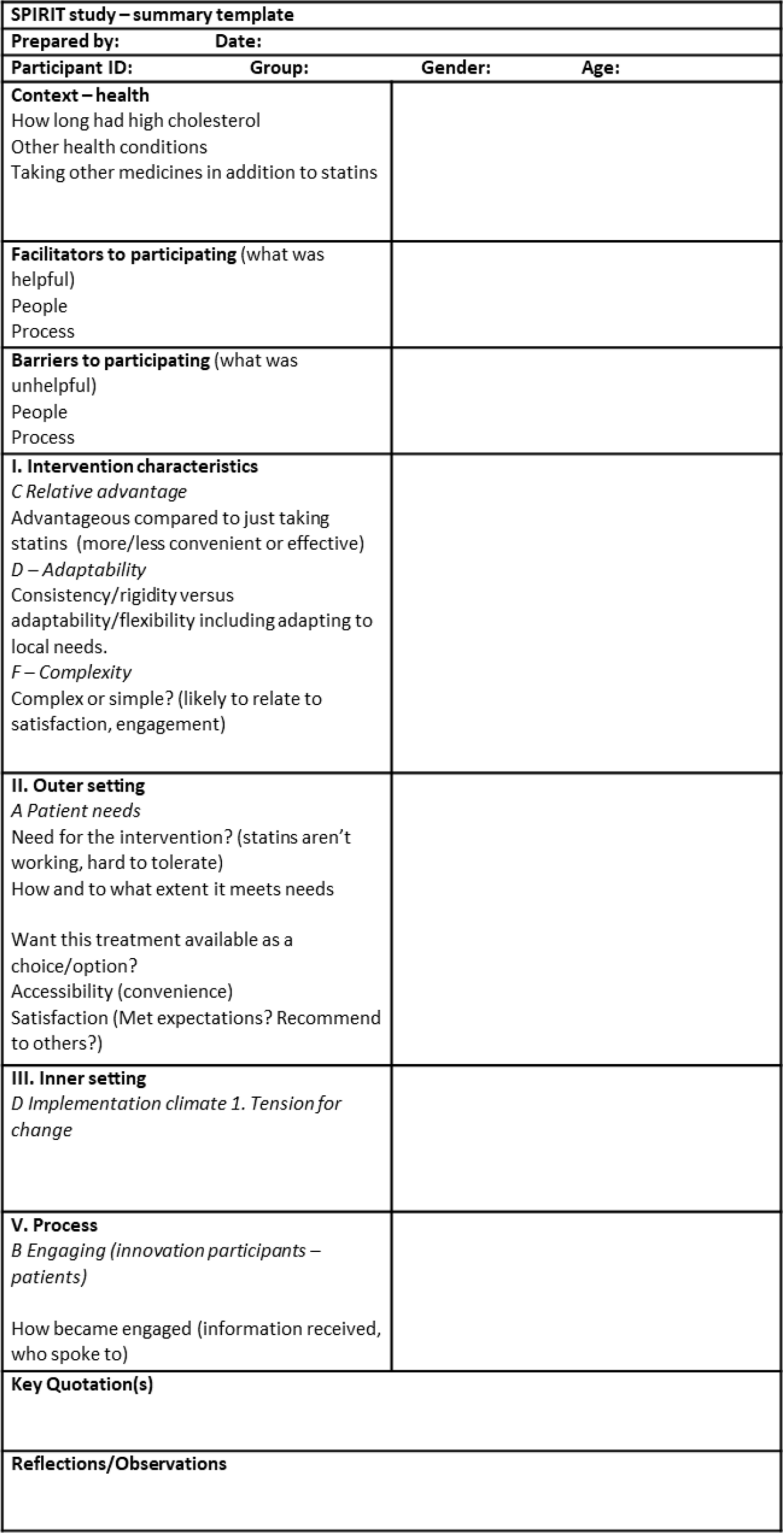
Finalised summary template for patient interviews.

Once drafted, the summary template was reviewed as a team and then independently tested by AM and RE after conducting pilot interviews. During testing, we made a number of minor changes to the templates including dividing one section into two (‘facilitators and barriers to participation’) to help the researchers organise the data. The process evaluation team lead (PW) reviewed the finalised templates before implementing them (step 3). Figure 2 is the finalised template used to summarise each patient interview.

### Step 3: completing the template soon after each interview

After each interview, the researchers (AM and RE) completed the summary template table in MS Word. While Hamilton’s and Gale et al’s approaches relied on transcribed data to generate a summary, we used field notes made during and shortly after the interviews. We aimed to complete the summaries as soon as possible after conducting the interviews, usually within 24 hours. Using field notes allowed us to expedite the process of starting data analysis before the transcripts were available. The ‘Observation’ box in the summary template was used to record researcher reflections (eg, ‘this is the youngest person we have interviewed so far and their experience seems unique’). We also noted in the summary template if a question was not asked or an answer pertaining to a particular CFIR domain or question was not given. After completing the summaries, the process evaluation team discussed each interview (step 4), and where necessary, reviewed the summary by listening to the interview audio. Listening to the interview resulted in minor changes to the summary and the addition of ‘key quotations’.

### Step 4: discussing analysis as a research team

After each interview, the research team (AM and RE) debriefed, in which we discussed details of the data and planned for future interviews. The research team also met virtually once a week, to discuss data collection and analysis; and the wider research team (AM, RE and PW) met virtually once a month. These meetings were useful as data collection and analysis were iterative, and they allowed the team to discuss potential themes and areas to explore in subsequent interviews. The debriefings and team meetings were also useful to establish consistency between the two researchers, with regard to how the summary templates were completed.

### Step 5: transferring summaries (step 3) to a matrix

Step 5 of the RA approach involved transferring the interview summaries (step 3) to a matrix in MS Word to identify reoccurring themes and compare findings across cases. To create the matrix, we used the CFIR domains (eg, intervention characteristics, inner setting and process), interview questions and themes (eg, ‘facilitators’ and ‘barriers’) identified in team discussions (step 4). The CFIR domains, interview questions and themes formed the columns in the matrix, and the individual participants formed the rows. Interview summaries (step 3) were used to populate the matrix. Summaries were reviewed multiple time by both researchers (AM and RE) independently before data were condensed in the matrix (table 1). The process evaluation team met virtually to discuss themes and subthemes.

**Table 1:** Summary template for patient interviews

ID(Anonymised ID (trial arm) gender, age, ethnic group)	Context	Facilitators(People)	Process	Barriers (People)	Process	Intervention characteristics (advantage, adaptability, complexity)	Outer setting - patient needs(Want as an option? Accessibility (convenience) satisfaction (met expectations? recommend?))	Inner setting(Is there a need for it?)	Process(Engagement (information received, who spoke to))	Other/notes
P02 (03) female, 70, white	Takes a statin, no issues, takes other medicines.	Found everyone helpful at injection.Finds behavioural support coaches helpful.	Convenient appointments overall (time, travel).Likes the behavioural support materials.Behavioural support appointments convenient, right amount and length.		Provided with a large volume of information – this was good.Appreciates style of behavioural support, not too directive.	Both injection and behavioural support are simple.	Abdominal injection is slightly more intrusive than having in arm.Receiving blood results was interesting but not essential.Would recommend to others.		Grateful for the NHS, keen to contribute to trial to potentially help self and others.Discussed participation briefly with husband but pretty much decided herself.	Little to say about injection, keen on behavioural support.
P07 (02) male, 78, white	Takes a statin for HC, no other health problems. HC remains raised.Follows a Mediterranean diet in attempt to control cholesterol.GP was going to give injection but got delayed with patient so nurse came and did it.	Pleasant atmosphere at appointment, chatted with the GP and found a family connection.Commented on nurse’s skill at administering injection (better than him when he’d self administered).	Convenient appointment overall – time, travel.	There were ‘others’ in the room, but didn’t know exactly who they were (although he didn’t seem bothered about this).		The intervention is simple.	Could be a good way of delivering treatment, if nurse-led may save moneyMet expectations expected minor discomfort from injection.Would recommend to others.	Needed, especially for people for whom statins are not effective in lowering HC.	Main motivation to participate was to get more monitoring with cholesterol – and hopefully lower it.Received letter and when practice phoned he was happy to take part.Discussed briefly with his wife.	Thinks nurses better than GPs at giving injections, also well placed and cheaper.
P56 (02) male, 55, Asian	High cholesterol diagnosed about 6 years ago, takes atorvastatin, otherwise healthy. HC is statin-resistant. Father died of heart attack.	People were helpful and the appointments were a nice environment, everything was explained well and they ‘had a good laugh’ too.	Everything has gone smoothly.	There were several people in the room, he didn’t know what they were doing although he doesn’t seem to have minded – anonymous.		Not had injection like this (into abdomen) before but it was fine.Day 90 showed lower cholesterol, so it’s working better than statins.Intervention is simple.	Convenient location and appointments.Would be happy to receive at the practice in the future.		Received a phone call and happy to take part.	Optimistic it would benefit him if he continued with it.Happy to take part and help out.

### Step 6: using the summary matrix to share findings

Finally, we used the summary matrix to inform presentations to key stakeholders, including the AHSN and the VICTORION-Spirit trial clinical team. Data from step 5 of the RA approach was also used to deliver an interim report for the study sponsor and funder, which summarised the barriers and enablers to the delivery of inclisiran and presented patients’, providers’ and commissioners’ views on future provision.^30^

## Results

Data collection for the process evaluation took place between August 2021 and April 2022. We conducted interviews with 56 patients across 17 participating general practitioner practices. 28 providers and eight commissioners were also interviewed. Data collection and analysis were conducted in parallel to share emerging findings in real time (‘Step 6’). 92 people participated in interviews; tables2  3 below summarise our sample.

**Table 2:** Patient participants

Characteristic	Inclisiran (n=28)	Inclisiran+behavioural support (n=28)
Sex		
Male	16	13
Female	12	15
Ethnic group		
White	22	24
Asian	4	1
Mixed	0	2
Ther	2	1
Age range	48–80	35–79

**Table 3:** Provider and AHSN participants

Interview group	Interviewees (n)
Healthcare providers	
Health professionals at participating practices	13
Health advisors	4
Research nurses	11
AHSN representatives	8

AHSNAcademic Health Science Network

### Completing the template soon after each interview

We found, depending on the length of the interviews (which ranged from 5 to 73 min), summarising took no longer than 2 hours. Summarising the interviews was quick and straightforward, particularly with some practice, as the table corresponded with the interview guide questions. Some summaries were longer than two pages, particularly if quotations were included. Overall, the time taken to generate the summary was considerably less than transcribing the interview data. Summaries were therefore available to the whole team sooner than interview transcripts. Further time savings were made by using field notes to complete the summaries rather than waiting for transcriptions.

The generated summaries were concise, succinct and relevant. Data from the interviews were mapped immediately to the CFIR domains, and key points were summarised. Once produced, the summaries superseded field notes, as they provided a record of the researchers’ reflections and observations, thus acting as memos. Regular debriefing and meetings as a research team to discuss the summaries also initiated comparison between ‘cases’, which we were able to document in subsequent summary templates.

### Discussing analysis as a research team

Completing the template generated a written summary that was available to the whole team soon after each interview. The interview summaries prompted discussion among the research team, which informed data collection and analysis. We also established inter-rater reliability by meeting virtually and discussing the RA summaries. This was less time intensive than ensuring consistency with how transcripts were coded.

### Transferring summaries to a matrix

Similar to the template, the matrices corresponded with the interview guides, which were informed by the CFIR, and were an effective way of summarising a large volume on data. Condensing the summaries into the matrix was straightforward. However, it was an iterative process, and when populating the matrix, we returned to the transcripts to verify details and identify examples of specific themes; particularly when the researcher was condensing summaries they had not produced themselves.

The matrices were useful to compare data across cases and identify key findings within the narratives. As a new innovation, it was particularly important to be able to convey views on acceptability and delivery from those individuals that the innovation was intended to benefit. Matrices were clear and understandable, and more accessible than the summaries when producing the interim report. The findings condensed in the matrix provided useful insights into patients’ experience of receiving the study treatment, which we were able to share with stakeholders, including patients’ experience of arranging and attending inclisiran injection appointments (CFIR domain *‘Process’*), and patients’ acceptability compared with daily statins (CFIR domains ‘Relative advantage’ & ‘Knowledge and beliefs about the intervention’). Analysis also identified patient barriers, mostly related to trial conduct, including wording on the participant information sheet; more people than necessary at the appointment; and the large volume of trial paperwork.

### Using the summary matrix to share findings

We found RA improved efficiency, as we were able to feedback barriers to stakeholders in real time via presentations. Overall, the presentations of the process evaluation findings were well received, and members of the trial clinical team commented that the findings validated their experiences. Attendees were keen for further updates. Comments and questions after the presentations also informed our inquiries, specifically sampling of the trial patients for participation in the process evaluation.

As discussed, the VICTORION-Spirit study took a novel approach to explore how best to deliver inclisiran within primary care, and inclisiran was introduced through the first NHS population health agreement between NHS England and Novartis Pharmaceuticals UK Limited—the study sponsor and funder. There was therefore a policy desire to share timely insights with implementers, both locally and nationally. Our RA approach allowed us to share timely findings from the process evaluation. Data collection and rapid analysis were conducted in parallel (between August 2021 and April 2022), which facilitated the sharing of interim findings with key stakeholders as the work progressed.

While our RA framework was less resource intensive than traditional methods, we found that after presentations to stakeholders, and when writing the interim report, we spent some time reviewing interview transcripts to identify quotes to illustrate findings. This added some time. However, because we had become so familiar with the data via debriefs, by meeting as a team to discuss analysis, and by transferring the summaries to the matrices (step 5), this did not take as long as anticipated. Moreover, interpretation and write up using RA appeared quicker than when using traditional methods.

## Discussion

In this article, we aimed to describe the qualitative RA technique and tool we developed and applied in the process evaluation for a phase IIIb hybrid trial. Furthermore, we sought to reflect on our experience of analysing and sharing findings in real time. Our experience suggests RA techniques can be useful within studies where there is a need to feedback to stakeholders and adjust implementation strategies accordingly in real time. Thus, supporting successful implementation efforts and accelerating adoption.

After reviewing the literature, Vindrola-Padros and Johnson^12^ made a number of recommendation for future use of RA techniques, including its applicability for applied research and its use to generate consensus among diverse participant groups. Our use of a RA framework is an example of how it can be used in applied health research, and how it helps to produce a coherent narrative among a diverse group of stakeholders, often with differing viewpoints. Our RA approach was consistent with the project’s requirement to produce information to inform ongoing implementation. It was therefore an appropriate and valid methodology, which produced robust findings on the barriers and enablers to the delivery of inclisiran. We were able to feedback these findings to the key stakeholders responsible for supporting the roll out of inclisiran, and the wider primary care community. Feedback from these stakeholders suggests our presentations were well received and useful. Similarly, Gale *et al*,^14^ when comparing analytic methods from a process evaluation of academic detailing in the Veterans Health Administration, found RA to be sufficient in providing actionable findings to their operations partner. Rapid analysis is therefore a useful technique, and worth considering in future applied health research, particularly if the project is constrained by short time frames.

Most of the articles reviewed by Vindrola-Padros and Johnson recommended the use of RA techniques for the reported benefits, including reduced cost, increased amount of collected data, improved accuracy and obtaining a closer approximation to the narrated realities of research participants. For our team, the main benefits to using our RA technique were the time saved and improved efficacy. Completing the summary template and condensing it into a matrix was quicker than transcribing and coding the data. Furthermore, while interviews were transcribed for future analysis, by relying on field notes, we could have saved transcription costs.

Despite highlighting the benefits to using qualitative rapid analysis techniques, some authors have discussed limitations and potential challenges. A key challenge surrounds maintaining trustworthiness given the rapid nature of the analyses. Moreover, many of the articles reviewed by Vindrola-Padros and Johnson^12^ that recommend rapid techniques also claim they cannot replace traditional methodologies. However, as Beebe^31^ said ‘rapid research’ is not the same as ‘rushed research’, and can still be rigorous. Not only did our RA approach save time, it also produced trustworthy findings by allowing us to collect data, engage in analysis and use theory in parallel to inform perspectives. Furthermore, our summary templates were useful for capturing complexity, while making the data easy to understand. Similar to member-checking techniques in traditional qualitative research, we were able to share these findings with the participants in a series of accessible presentations, and an interim report,^30^ to check the results resonance with their experiences. We also found debriefing and regular meetings as a research team to discuss analysis, and how we completed the summary templates, were crucial to the success of our RA approach and ensured trustworthiness. We would advise research teams considering using RA to incorporate this into their design.

It is acknowledged that the deployment of rapid techniques often requires the willingness from a team of experienced researchers to engage in new methodologies.^12 32^ The team’s experience grounded in traditional qualitative in-depth analysis undoubtedly helped, and the credibility of the findings was arguably enhanced by the team’s expertise in qualitative methods and implementation science. More work is needed to establish what training—other than learning how to create and complete the template—is required. Nonetheless, as Nevedal *et al*^32^ argued, our RA technique may be more suited to researchers who have a foundation in the CFIR or could be adapted to use other frameworks familiar to the research team.

There are advantages to using frameworks such as the CFIR in a rapid qualitative analysis approach. First, CFIR is a comprehensive framework, which incorporates pre-existing literature on the barriers and enabler of the adoption and spread of innovations. The research questions we aimed to explore were therefore well suited to the CFIR, and specifically, a deductive approach to help identify the determinants that may help or hinder the implementation of inclisiran. Second, use of the CFIR helped our team compare findings across cases. Overall, we conducted 92 interviews with a range of stakeholders, often with contrasting views. The use of the CFIR provided structure and consistency. However, as discussed, so as not to overly rely on the CFIR, we included questions from our interview guides beyond the CFIR domains in the summary template and included space for inductive topics. While the use of a rapid analysis approach, and specifically, use of the CFIR, depends on the aims and objectives of the study, we found our deductive approach was appropriate for this process evaluation. Further, with the emphasis on speeding up adoption of evidence-based interventions, and to support real-time policy decision making, we would argue future process evaluations should consider rapid methods. Specifically, teams could consider using our rapid analysis framework.

## Limitations

The main limitation of this study is that we did not compare our RA approach with a traditional qualitative analysis approach such as thematic analysis.^33^ Thus far only a handful of studies have compared analytical approaches.^13 14 32^ A key study comparing approaches is that of Taylor *et al*,^13^ in which they compared rapid and traditional analyses of a UK health service evaluation dataset. They found the rapid analysis took less time than thematic analysis (100 hours vs 126.5 hours). Similarly, Gale *et al*^14^ and Nevedal *et al*^32^ both reported time savings, with Nevedal *et al* also reporting transcription cost savings. Nevedal *et al* found their deductive CFIR approach required 409.5 analyst hours in total compared with 683 hours for their traditional deductive CFIR approach. Furthermore, Gale *et al* and Taylor *et al* both found consistency between their RA and in-depth analysis findings.

Our RA approach is similar to that of Gales *et al*^14^ and Nevedal *et al*.^32^ However, unlike Gale’s and colleagues’ approaches, we did not rely on transcribed data and instead used field notes. In addition, as recommended by Nevedal and colleagues to streamline their approach, we did not use a second analyst when creating the summaries (step 3), which saved time. Therefore, it is reasonable to assume we made similar time savings. Further, we would argue that our RA approach was less resource intensive than those of Gale *et al* and Nevedal *et al*, due to the aforementioned reasons.

## Conclusion

Our study developed and tested a novel application of RA, which not only saved time when compared with traditional analysis methods but also improved efficacy. We were able to share timely findings on what helped and hindered implementation of inclisiran with the study sponsor, and local and national implementers. This was particularly pertinent given the unique approach to implementation and a policy context that emphasised accelerated innovation adoption and spread. We hope, by reflecting on our experiences of applying this RA framework, other researchers might be able to incorporate it into their designs. Rapid analysis may provide a solution to the need to disseminate qualitative findings in short time frames. Specifically, our approach could facilitate the use of findings in implementation processes in policy and practice. However, further work is required to compare the RA approach to traditional qualitative methods with regard to time savings, training and rigour.

## Data Availability

Data are available upon reasonable request.
