# Supplementary material for: Development and application of a qualitative rapid analysis framework in a hybrid trial within primary care
Source: BMJ Open. 2024 Jul 24;14(7):e076792. doi: 10.1136/bmjopen-2023-076792 (PMC11284935; doi:10.1136/bmjopen-2023-076792)
Supplement: Supplementary file 1 [file bmjopen-2023-076792.reviewer_comments.pdf]

PEER REVIEW HISTORY

BMJ Open publishes all reviews undertaken for accepted manuscripts. Reviewers are asked to complete a checklist review form (**http://bmjopen.bmj.com/site/about/resources/checklist.pdf**) and are provided with free text boxes to elaborate on their assessment. These free text comments are reproduced below.

ARTICLE DETAILS

**TITLE (PROVISIONAL)**	Development and application of a qualitative rapid analysis framework in a hybrid trial within primary care
**AUTHORS**	Mathieson, Amy; Elvey, Rebecca; Wilson, Paul

VERSION 1 – REVIEW

**REVIEWER**	Jérémie Beaudin<br>Université de Sherbrooke, Faculté de Médecine et des sciences de la santé
**REVIEW RETURNED**	30-Aug-2023

**GENERAL COMMENTS**	Thank you for this opportunity to review this innovative work. Indeed, faster ways to speed up the qualitative analysis and dissemination processes are important matters to improve this field of research. After carefully reading your article, here are a few recommendations and comments for fine-tuning your work.<br><br>Background: This section is well written and easy to understand. I have no comment for this section.<br>Methods: This section is fairly brief and would benefit from more detail.<br>Please state what were the inclusion criterias for your participants<br>Please further describe your participants (n, characteristics)<br>How many participants were interviewed via telephone VS via video conferencing?<br>Please provide a short description of the researchers qualitative experience involved during data collection and analysis.<br><br>Data analyse approach: This section describing your approach is clear and comprehensible.<br>Step 3: Can you describe why you chose to field notes instead of using transcribed data to generate a summary (Hamilton and Gale's approaches)?<br><br>Step 6: The summary matrix is an interesting way to visualize data. Is there a way you can provide an example (as a figure, suppl. material) of this matrix?<br><br>Results, discussion: These sections provide great insights about your approach and its benefits , as well as limitations, and clarifies many things.<br><br>Thank you very much.

VERSION 1 – AUTHOR RESPONSE

Reviewer 1	
Thank you for this opportunity to review this innovative work. Indeed, faster ways to speed up the qualitative analysis and dissemination processes are important matters to improve this field of research.<br>After carefully reading your article, here are a few recommendations and comments for fine-tuning your work.	Thank you.
Background: This section is well written and easy to understand. I have no comment for this section.	Thank you.
Methods: This section is fairly brief and would benefit from more detail.<br><br>Please state what were the inclusion criterias for your participants<br><br>Please further describe your participants (n, characteristics)<br><br>How many participants were interviewed via telephone VS via video conferencing?<br><br>Please provide a short description of the researchers qualitative experience involved during data collection and analysis.	We have added detail in the following sections:<br><br>Method, page 10, line 11 onwards, added text.<br><br>We have added two tables summarising the participant characteristics to page 18<br><br>Results, page 10, line 16, added text.<br><br>Method, page 10, line 6, added text.
Data analyse approach: This section describing your approach is clear and comprehensible.<br>Step 3: Can you describe why you chose to field notes instead of using transcribed data to generate a summary (Hamilton and Gale's approaches)?	This was to expedite the analysis process, instead of waiting for transcripts to be returned from the transcriber.<br><br>We have added text describing this on page 15, line 10.

Step 6: The summary matrix is an interesting way to visualize data. Is there a way you can provide an example (as a figure, suppl. material) of this matrix?	Thank you, yes we found it a useful way to visualize data. Thank you for the suggestion – we have added a figure that shows the columns with a few rows from the patient summary matrix.<br><br>This will be figure 3, which we suggest would fit best as a supplementary file due to its size, we have inserted signposting text at an appropriate place on page 16.
Results, discussion: These sections provide great insights about your approach and its benefits , as well as limitations, and clarifies many things.	Thank you.

VERSION 2 – REVIEW

**REVIEWER**	Jérémie Beaudin<br>Université de Sherbrooke, Faculté de Médecine et des sciences de la santé
**REVIEW RETURNED**	07-Mar-2024

**GENERAL COMMENTS**	Thank you for your revision. I have no more comments for the authors.
